# An Automated Mobile Mood Tracking Technology (Mood 24/7): Validation Study

**DOI:** 10.2196/16237

**Published:** 2020-05-20

**Authors:** Anupama Kumar, Michael Wang, Alison Riehm, Eileen Yu, Ted Smith, Adam Kaplin

**Affiliations:** 1 Department of Psychiatry and Behavioral Sciences Johns Hopkins University Baltimore, MD United States; 2 Department of Pharmacology and Toxicology University of Louisville Louisville, KY United States

**Keywords:** depression, text messaging, patient monitoring, mobile phone, short message service, ecological momentary assessment, digital health

## Abstract

**Background:**

Electronic tracking has been utilized for a variety of health conditions. Previous studies have shown that there is higher adherence to electronic methods vs paper-and-pencil tracking modalities. Electronic tracking also ensures that there are no back-filled entries, where patients have—to appear compliant—entered their responses retrospectively just before their visits with their health care provider. On the basis of the recognition of an unmet need for a Web-based automated platform to track psychiatric outcomes, Johns Hopkins University partnered with Health Central (a subsidiary of Remedy Health Media LLC) to develop Mood 24/7, an electronic, mobile, automated, SMS-based mood tracker. This is a pilot study to validate the use of Mood 24/7 in anticipation of clinical trials to demonstrate the therapeutic benefit on patients’ health outcomes of utilizing digital mood-tracking technology.

**Objective:**

Mood 24/7 is an electronic mood-monitoring platform developed to accurately and efficiently track mood over time through automated daily SMS texts or emails. This study was designed to assess the accuracy and validity of Mood 24/7 in an outpatient psychiatric setting.

**Methods:**

This pilot study involved a retrospective chart review for depressed outpatients (N=9) to compare their self-reported Mood 24/7 daily mood ratings with their psychiatrist’s independent clinical mood assessment at the time of the patient’s visit. Their mood ratings via Mood 24/7 were collected over 36 weeks. In addition, a mixed model analysis was applied to compare the weekly Montgomery-Åsberg Depression Rating Scale (MADRS) scores with Mood 24/7 scores over an average of 3 months.

**Results:**

A 97.2% (315/324) digital mood reporting adherence was found over 36 weeks, and a significant correlation (*r*=0.86, *P*<.001) was observed between patients’ Mood 24/7 scores and their psychiatrist’s blinded clinical assessment of the patient’s mood when seen in the clinic. In addition, a significant concordance (intraclass correlation of 0.69, 95% CI 0.33-0.91, *P*<.001) was observed in the mixed model analysis of the clinician-administered MADRS vs Mood 24/7 scores over time.

**Conclusions:**

Our chart review and mixed model analyses demonstrate that Mood 24/7 is a valid instrument for convenient, simple, noninvasive, and accurate longitudinal mood assessment in the outpatient clinical setting.

## Introduction

### Background

The virtually universal adoption and use of personally accessible technology have transformed the way patients monitor their health and medically relevant signs and symptoms over time. Electronic tracking has been utilized for a variety of health-related conditions, including pain [1], migraine [2], gastroesophageal reflux [3], and cancer [4], to name just a few. There are many benefits to electronic health tracking and reporting, as patient-reported ease of use and satisfaction are generally higher in digital tracking vs analog (eg, pen and paper) tracking [1,5]. Furthermore, the vast majority of studies conducted to compare analog vs digital health outcome tracking report higher adherence using electronic methods [6].

Low patient adherence to the temporal requirements of routine paper record entries and the possibility of patients backfilling entries to compensate for missed days pose significant problems with paper tracking methods. To measure the difference in patient-reported, handwritten diary adherence, a 2003 study embedded photosensors into paper diaries to track the opening of the diary [7]. Patient-reported paper compliance rates were 90%, but analysis of photosensor data gave an actual compliance rate of only 11%, demonstrating a high level of nonadherence and embellishment among patients. This study also included a patient group that used electronic diary tracking, where the patient was aware that all entries were time-stamped. As compared with the 11% actual adherence observed in written diary tracking, that of electronic diary tracking was dramatically higher at 94%. Taken together, these findings suggest that patients are more likely to adhere to regular recordings using digital technology compared with paper records. With this in mind, along with the recognition of an unmet need for a Web-based automated platform to track psychiatric outcomes, Johns Hopkins University partnered with Health Central (a subsidiary of Remedy Health Media LLC), who developed and maintained Mood 24/7.

### Objective

Mood 24/7 is an electronic mood monitoring system that offers users the flexibility to track their mood via the technology they already own and carry around on a daily basis: their mobile phone. Users sign up on the Web [8] by registering their basic contact (name, phone, email), and specifying what time of day they would like Mood 24/7 to send them an SMS text message inquiring about their mood. No apps are needed or used as part of Mood 24/7, and instead, it is run via a Web-based secure service employing SMS texting for registration and communication. A wireless service sends the daily text message to the patient, asking the patient to rate their mood using a standard Likert scale: *On a scale of 1 (low) to 10 (high) what was your average mood today?* Daily mood ratings (and annotations of unlimited length and frequency, if entered) are compiled into a mood chart stored on a Health Insurance Portability and Accountability Act-compliant server that a patient can access by logging onto their encrypted Mood 24/7 account at any time. Moreover, the patient can choose to share their mood chart with any number of their health care providers or friends and family, making it a real-time mobile electronic health diary.

This study was designed to assess how well patient self-assessments of their mood over time on Mood 24/7 correlates with traditional methods of evaluating patients’ mood and depression, including clinician ratings and the Montgomery-Åsberg Depression Rating Scale (MADRS) [9].

## Methods

### Subjects

A total of 9 sequentially chosen outpatients (4 women) met the inclusion criteria for the study, which consisted of (1) a diagnosis of major depressive disorder and (2) agreement and ability to use Mood 24/7 (essentially any mobile phone with SMS texting capability). Exclusion criteria included all moderate to severe substance use disorders except tobacco. These patients were followed for at least 36 weeks. All protocols were approved by the Johns Hopkins University Institutional Review Board.

### Clinician Mood Rating

The clinician mood ratings were the clinical evaluations by the psychiatrist upon completion of the patient’s office visit based on a 1 to 10 mood scale, with 1 corresponding to the worst mood state the patient ever had, and 10 a return to euthymia. The psychiatrist was blinded to the patient’s Mood 24/7 self-ratings.

### Mood 24/7

All patients signed up for Mood 24/7 at www.mood247.com. The daily mood was tracked via the patient entering their mood rating between 1 and 10 in response to the following daily prompt occurring at a flexible single daily time of their choosing: *On a scale of 1 (low) to 10 (high) what was your average mood today?*

### Statistical Analyses

**Figure 1:**
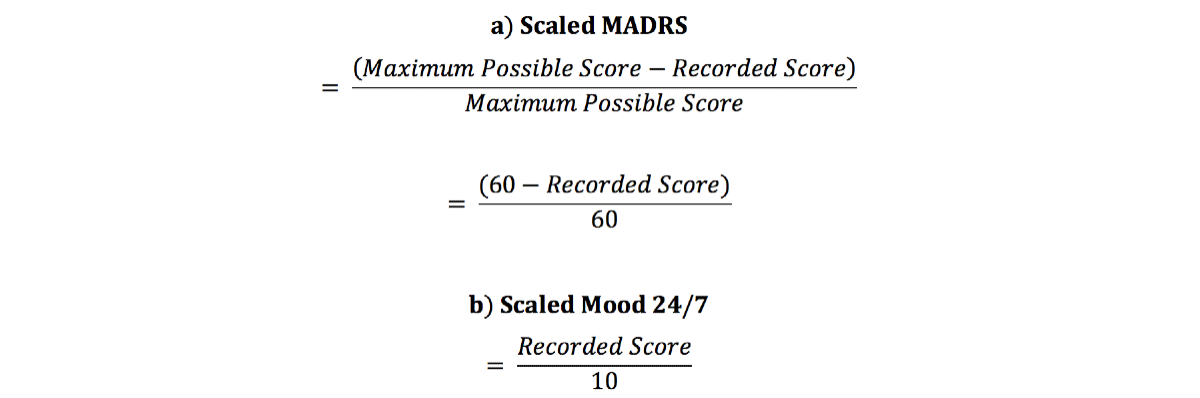
Montgomery-Åsberg Depression Rating Scale (MADRS) (a) and Mood 24/7 (b) scores were converted to a common scale (0 to 1). Note that MADRS scaling was also inverted such that improvements in mood were represented as greater values.

Mood 24/7 ratings were correlated with clinical rated scores. Statistical analyses were conducted using GraphPad Prism 5.0 (GraphPad Software LLC). Two-tailed Pearson correlation analyses were conducted between Mood 24/7 ratings and clinician mood ratings. *P* values <.05 were considered statistically significant. Absolute adherence of these patients to Mood 24/7 was determined by counting the number of weeks they responded without interruptions. Interruptions were considered as 7 consecutive days without a single response. Adherence was considered as at least one response per week [10]. Absolute adherence of the 9 patients over 36 weeks was calculated by taking the ratio of the number of weeks without interruption divided by the total number of weeks. To further test the validity of Mood 24/7 against a gold-standard assessment [11], a linear mixed effects model was used to compare the concordance of Mood 24/7 reports and MADRS longitudinal assessments using STATA 14 (Stata Corp). Mood 24/7 scores of 3 patients with treatment-resistant depression were used, with time and patient number as fixed effects. This method is robust against missing data, which could be the result of a patient omitting a report on a given day or a missed clinician assessment. For improved intelligibility of time-series data comparing changes in daily mood ratings vs MADRS assessments, scores were transformed to a common scale using the following formula in Figure 1

The effects in the mixed model were determined by the likelihood ratio test.

## Results

### Clinician Mood Rating Correlated with Mood 247

**Figure 2:**
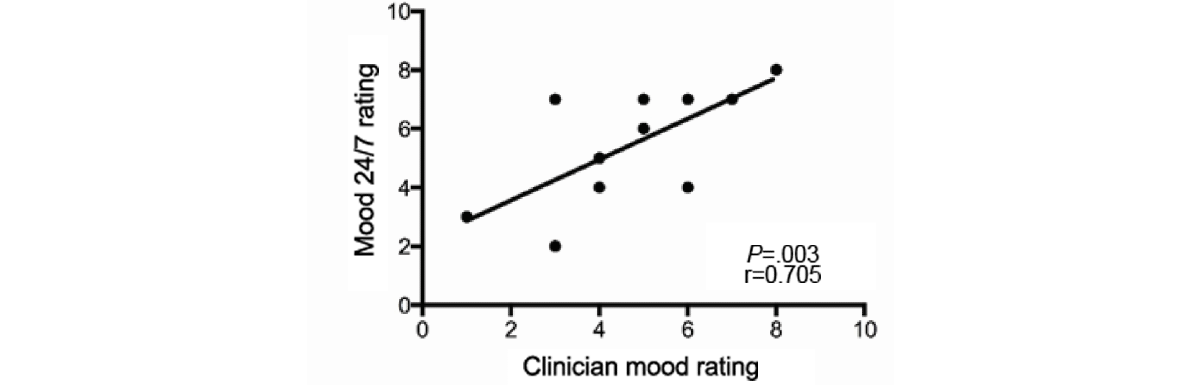
Validation of Mood 24/7. Both the mood ratings assigned by the clinician and mood determined by a standard mood assessment test correlate with patients’ Mood 24/7 self-assessment rating. Clinician mood ratings positively correlated with Mood 24/7 rating (*P*=.003).

A clinician’s assessment of their patient’s mood was correlated to the patient’s self-reported Mood 24/7 scores from the same day, and a significant positive correlation was observed between the clinician mood rating and Mood 24/7 rating (*r*=0.705, *P*=.003; Figure 2). To further corroborate the use of Mood 24/7 as an accurate and reliable means to track patient mood over time, clinician mood ratings were plotted against mood data collected across multiple days from 9 consecutive independent patients.

### Clinician Mood Rating

**Figure 3:**
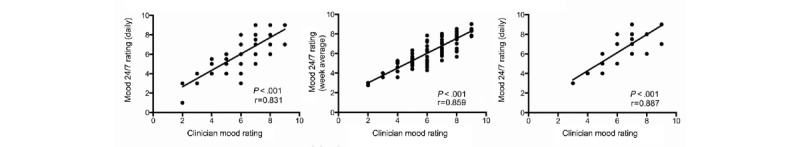
Long-term validation of Mood 24/7. Data from 9 patients collected over multiple office visits demonstrate lasting correlations between clinicians’ mood assessment and patients’ Mood 24/7 self-assessment rating. Positive correlations were found between the clinician mood rating and daily Mood 24/7 rating of 100 data points, with each point representing a unique patient and clinician assessment (*P*<.001; left). Similarly, positive correlations were found between the clinician mood rating and the weekly average of Mood 24/7 rating of 100 data points (*P*<.001; center); 44 data points collected from 1 patient over the course of 2 years demonstrated strong positive correlations between the clinician mood rating and Mood 24/7 rating (*P*<.001; right).

Patients received a clinician mood rating (on a 1-10 scale) at every office visit (average=11 visits per patient, over an average length of follow-up of 36 weeks). Values were correlated to both the patient’s Mood 24/7 self-rating given on that day (Figure 3, left graph) and the patient’s Mood 24/7 weekly rating obtained by averaging the mood scores from 3 days before, the day of, and the 3 days after their clinic visit (ie, the week average, Figure 3, center graph). This was done to account for small variations potentially introduced by the effect on a patient’s mood after seeing their treating psychiatrist. Significant correlations were reached for daily (*r*=0.83) and weekly averaged (*r*=0.859) Mood 24/7 ratings, after controlling for the number of office visits per patient and frequency of office visits (*P*<.001). Data analyzed from 1 patient who used Mood 24/7 for the longest duration (approximately 2 years, with a daily adherence rate of 84%) showed that the Clinical Mood Rating and Mood 24/7 self-rating correlation (*r*=0.887) was maintained over time (Figure 3, right graph; *P*<.001). The weekly adherence of the 9 patients to Mood 24/7 over 36 weeks was 97.2% (315/324), and daily response adherence was 60.60% (1386/2286; with the least responsive at 93/254, 36.6% and the most at 214/254, 84.3%).

### Montgomery-Asberg Depression Rating Scale

**Figure 4:**
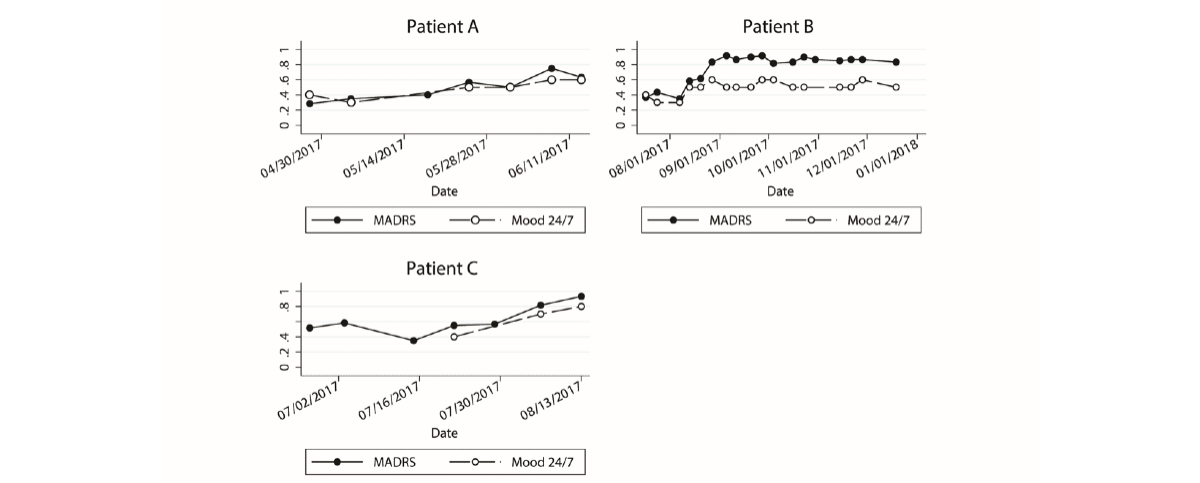
Scaled Mood 24/7 and Montgomery-Åsberg Depression Rating Scale (MADRS) scores over 3 months. A mixed effects model analysis was applied to the longitudinal data shown here. Scaled MADRS and Mood 24/7 ratings are shown by the patient. Data points represent clinician or patient mood scores for that given day. Mixed model analysis indicates significant concordance between Mood 24/7 and MADRS scores within each patient (*P*<.001), and postestimation analysis showed an intraclass correlation of 0.69.

Another method we used to strengthen the validation of Mood 24/7 was to compare changes in weekly MADRS and Mood 24/7 mood scores in a group of 3 patients with treatment-resistant depression (ie, failure to respond to at least two adequate antidepressant trials, and who were newly placed on another treatment regimen). A mixed model analysis was performed on these patients’ MADRS assessments by a trained mental health practitioner and the patient’s Mood 24/7 ratings. MADRS is a 10-item diagnostic questionnaire administered and scored by a trained rater with mental health experience to measure the severity of depression. The patients’ MADRS scores were compared with their Mood 24/7 scores over an average of 3 months (Figure 4). A mixed model analysis, with patient number and time as fixed effects, demonstrated high concordance between Mood 24/7 and MADRS scoring over time. The postestimation analysis of the mixed model demonstrated an intraclass correlation of 0.69 (CI 0.33-0.91) and *P*<.001 between clinician-rated MADRS and patient-selected Mood 24/7 scores.

## Discussion

### Principal Findings

This study was designed to validate Mood 24/7 as a Web-based mood-tracking technology for convenient, accurate, and reproducible monitoring of patients’ mood between clinic visits. This was a pilot study for validation as a mood tracking tool in anticipation of subsequent studies of therapeutic efficacy and improved patient health outcomes. Here, we demonstrated strong and significant correlations between patients’ self-reported Mood 24/7 scores and their performance on the MADRS. MADRS is a well-established standardized test for mood routinely used to evaluate depression [12]; it is the second most widely used depression rating scale, has high interrater reliability, and correlates significantly with the Hamilton rating scale for depression [13]. Our findings demonstrated that patients self-ratings are on par with the sensitivity of the established and previously validated MADRS, indicating that Mood 24/7 can provide accurate mood data for the purposes of monitoring a patient’s level and severity of depression over periods of time when the MADRS cannot be administered (ie, between office visits).

The mixed model analysis of Mood 24/7 scores vs MADRS assessment demonstrated that Mood 24/7 is a valid instrument for noninvasive and accurate mood assessment and depression tracking. Most notably, this preliminary analysis, based on a 2016 meta-analysis that recommends a correlation of 0.4 to 0.7 between self-report and clinician reports on a mood rating scale, indicated that Mood 24/7 is a robust instrument for longitudinal collecting and reporting of a patient’s mood [14].

Although scores were demonstrated to be concordant using the mixed model analysis, absolute scores between Mood 24/7 and MADRS were not linearly related, and there were notable ceiling and floor effects in patient self-reporting. These effects can be attributed to idiosyncratic patient preference, which is common to all subjective estimation activities. A similar phenomenon was observed in comparing the clinician’s and patient’s rating scores (see Figure 3, center graph): (1) when a patient is clearly depressed (eg, mood<5) or clearly euthymic (eg, mood>9), there is high concordance in the absolute scores given, but (2) what a patient considers a mood score in the middle of the range (eg, 5-7) may differ by 1 or 2 points from what the clinician estimates their mood. Thus, although patients vary in their subjective definition of their absolute mood rating, the changes over time in their Mood 24/7 are indicative of changes in their level of depression.

The results demonstrated keen mood self-awareness and sustained adherence by patients, even during periods of significant levels of depression. Mood 24/7 correlations with clinician ratings remained strong over a long period (ie, multiple years) of follow-up treatment, supporting the use of Mood 24/7 as a longitudinal outcome assessment and treatment monitoring tool. Taken together, these analyses exhibit the accuracy and reliability of Mood 24/7 in outpatients being treated for depression.

Slightly stronger correlations were observed between the clinician assessment of patient mood and the weekly patient Mood 24/7 ratings as compared with daily patient Mood 24/7 ratings. It is possible that the patients’ daily Mood 24/7 rating on the day of their clinic visit was affected by their visit, thus providing more variability and accounting for the somewhat weaker correlation. For example, if a patient is very apprehensive about a psychiatric appointment and receives their Mood 24/7 text prompt before the visit, then he or she may rate their mood lower than usual. Conversely, if a patient is reassured and pleased with his or her psychiatric appointment, then the patient might rate their mood higher than usual when sending their text response to Mood 24/7. In this case, and for future studies, a weekly average mood rating might provide a more accurate representation of actual mood as compared with one mood value from the day of the clinic visit.

This study offers two findings that are of importance to the population with depression. First, changes in Mood 24/7 measurements are valid when compared with the trained clinician’s evaluations. Second, this approach is associated with high patient adherence over time [15]. There are other technology platforms that offer patient self-rated mood reporting that differ along dimensions relevant to adherence. Electronic diaries such as Medicus or Actiwatch have been utilized to track mood and other psychiatric outcomes, but these diaries require a stand-alone electronic device [16,17]. Mood 24/7 allows for continuous and daily monitoring of the patient’s mood by utilizing the mobile device they already possess—any phone that can receive and send an SMS text is sufficient. Between appointments with the clinician or therapist, both the patient and health care professional can, but do not have to, check the daily mood ratings on the Web and in real-time by logging in to their private account at www.mood247.com. The patient can also include family, friends, or other health care professionals into their *trusted circle*, a feature of Mood 24/7 that enables those included in the circle by the patient to log into the patient’s mood chart, enter their own observations, and track the mood of the patient remotely. Thus, Mood 24/7 facilitates collaborative and potentially multidisciplinary care coordination. Family and friends can be given peace of mind by regularly checking up on an individual who has invited them into their trusted circle without having to directly contact the patient and inquire about their mood. Patient response to the daily reporting-style question utilized in Mood 24/7 is time- and date-stamped. Data cannot be entered retrospectively, which prevents any recall bias [18]. The adaptation and implementation of Mood 24/7 for use in clinical trials are straightforward. Finally, the text-based use of Mood 24/7 is for US citizens. Mood 24/7 uses text messages to reach users instead of a mobile app, so it is not limited to smartphone owners. For those living outside of the United States (or for those in the United States who prefer email over text messaging), the Mood 24/7 prompt can be sent via a daily email with a link that directs patients to a secure (eg, https) data entry site.

Mood 24/7 has the potential to improve quality of care for patients in many areas of health care beyond mood-related disciplines. For example, the Likert scale is considered to be the *gold standard* for sleep quality assessment [19]. The texting technology and Likert scale–style question employed in Mood 24/7 could be easily adapted to the longitudinal monitoring of sleep, anxiety, sexual function, memory, concentration, and pain [10].

### Limitations

There are several limitations to this study. The results might have been affected by the small sample size of 9 patients in the first study and 3 patients in the second study. However, the strong correlations observed, despite the relatively small sample size, suggest that Mood 24/7 is a highly sensitive and precise tool that aligns with a traditional depression outcome measure (MADRS and clinician ratings). Moreover, with respect to the 9 subjects who were followed for 36 weeks, this amounts to a total of 2268 daily texts, 324 weeks, and over 6 patient-years of follow-up. The 3 patients who underwent weekly MADRS assessments were followed for an average of approximately 80 days or over 11 weeks. Another possible source of variability in this study could be the timing of the Mood 24/7 text message. Patients program the Mood 24/7 software to send a text at the same time every day, asking them to gauge their average daily mood, so variability in mood throughout the day, particularly in the bipolar patients, might be more difficult to capture. This needs to be tested empirically.

The mixed model analysis was limited to 3 patients undergoing intensive psychiatric assessment in the setting of severe treatment-resistant depression. In addition, all MADRS assessments were performed by the same two clinicians. The preliminary significance of these results warrants further study in a larger population to better understand the potential utility of Mood 24/7 across a large population of diverse patients.

Although this pilot study did show that daily Mood 24/7 scores positively correlate with clinician assessments and MADRS scores, future studies will involve a larger patient population followed over a longer period of time. Another point that will be addressed in future studies is the probable therapeutic effect of daily mood tracking on the patients’ mood [20].

### Conclusions

In conclusion, Mood 24/7 has been shown to be a reliable and accurate predictor of both a standardized depression scale (ie, MADRS) and the clinician’s assessment of the patient’s mood. This study, designed to validate the use of Mood 24/7, provides support and rationale for the use of integrated mobile phones and Web-based technology to track and monitor mood and outcome of patients being treated for depression. Mood 24/7 has the potential not only to improve the quality of clinical studies that measure mood over time but also to improve the quality of an individual’s mental health care.

## Abbreviations

MADRS: Montgomery-Åsberg Depression Rating Scale
